# Comparison of clinical outcomes between sequestered cervical disk herniation and non-sequestered cervical disk herniation after anterior cervical decompression and fusion: a cohort study

**DOI:** 10.1186/s13018-023-04515-9

**Published:** 2024-01-05

**Authors:** Lanbo Jin, Ke Sun, Gang Liu, Wen Yuan, Huajiang Chen, Ye Tian

**Affiliations:** Present Address: Department of Orthopedics, Spine Center, Changzheng Hospital, Naval Medical University, 415 Feng Yang Road, Huangpu District, Shanghai, 200003 China

**Keywords:** Anterior cervical decompression and fusion, Cervical spine, Sequestered disk, Outcomes, Cord signal change

## Abstract

**Background:**

The advantages of anterior cervical decompression and fusion (ACDF) were well published, while research on postoperative results in different subtypes of cervical disk herniation (CDH) still remains blank. This study aimed to explore the surgical outcome between sequestration and other types in CDH.

**Methods:**

This retrospective cohort study enrolled 108 patients treated with ACDF in our hospital. The participants were divided into two groups according to the existence of a sequestered disk. The Visual analog scale score, the Japanese Orthopedics Association (JOA) score and the Neck disability index score were used to evaluate postoperative outcome.

**Results:**

Significant improvements were observed in both groups at every viewpoint (*P* < 0.001). The mean JOA was 15.04 ± 1.26 in the sequestered disk group and 14.45 ± 1.43 in the non-sequestered disk group two months after the operation (*P* = 0.026 < 0.05). The improvement in JOA at two months after ACDF showed a significant difference: 46.58% ± 39.17% in the sequestered disk group and 33.39% ± 28.82% in the non-sequestered disk group (*P* = 0.047 < 0.05). Thirty-two patients in the sequestered disk group (64%) and 19 patients in the non-sequestered disk group (32.76%) presented with high signal intensity of the spinal cord on preoperative cervical T2-weighted MRI (*P* < 0.001).

**Conclusions:**

Patients with sequestered cervical disks seemed to have a higher degree of symptom improvement two months after ACDF. CDH with a sequestered disk appears to be more likely to cause high signal intensity changes in the compressed cervical spine on T2-weighted MRI. We prefer early positive surgery in patients with sequestered cervical disks from the clinical point of view.

## Introduction

It has been verified that cervical disk herniation (CDH) can result in a series of syndromes, such as neck and shoulder pain, upper extremity tingling and numbness. In severe cases, it can lead to numbness of the lower limbs, walking instability, quadriplegia, impaired urinary function and other serious consequences [1]. The therapeutic effect is usually directly related to the degree of improvement in these symptoms. The symptoms of cervical spondylosis caused by CDH are largely due to the compression of the herniated disk on the contents of the spinal canal and nerve roots, which will lead to increasing pressure of the compressed spinal cord segment, and the intramedullary blood supply will be affected, resulting in local spinal cord cell edema, inflammation and myelomalacia.

In clinical research, referring to the classification of lumbar disk herniation (LDH), we can also perform a simple grading of the degree of CDH [2]. Three types of herniation ranging from mild to severe can be observed in cervical intervertebral disk [3]: protrusion, extrusion and sequestration. A bulging disk is not considered a form of herniation. Sometimes, an extruded disk is not well distinguished from a protruded disk. Extruded disk material that has no continuity with the disk of origin can be defined as “sequestrated.” Magnetic resonance imaging (MRI) is the most valuable method that can demonstrate the differences in these types [4].

The prevalence of CDH is still not clear. Meanwhile, a significant portion of patients who have received CDH have very mild symptoms. Moreover, regular follow-up observation and conservative treatment usually achieve good results in CDH [5]. However, in most cases of sequestered CDH, the fragments from diseased intervertebral disk usually compress the dural sac and cause severe spinal cord lesions. Timely and effective surgical treatment will lead to a better prognosis [1]. Anterior cervical decompression and fusion (ACDF) is the gold standard in case of symptomatic cervical disk herniation resistant to medical care [6, 7].

Scholars have performed many studies on the efficacy of cervical spine surgery [8]. However, researches on prognosis after surgery in different subtypes of CDH have not been reported. Therefore, we devised this retrospective cohort study to assess the operational effect between sequestration and other types in CDH.

## Materials and methods

### Design and participants

**Fig. 1:**
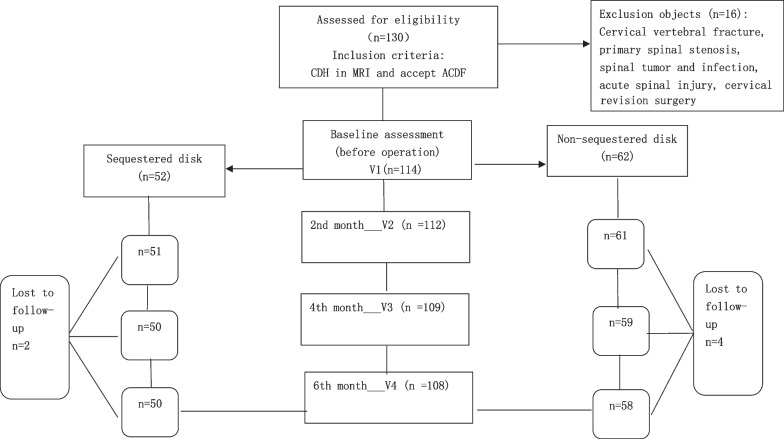
Flow diagram of the study. *CDH* cervical disk herniation, *ACDF* anterior cervical decompression and fusion, *MRI* magnetic resonance imaging

**Fig. 2:**
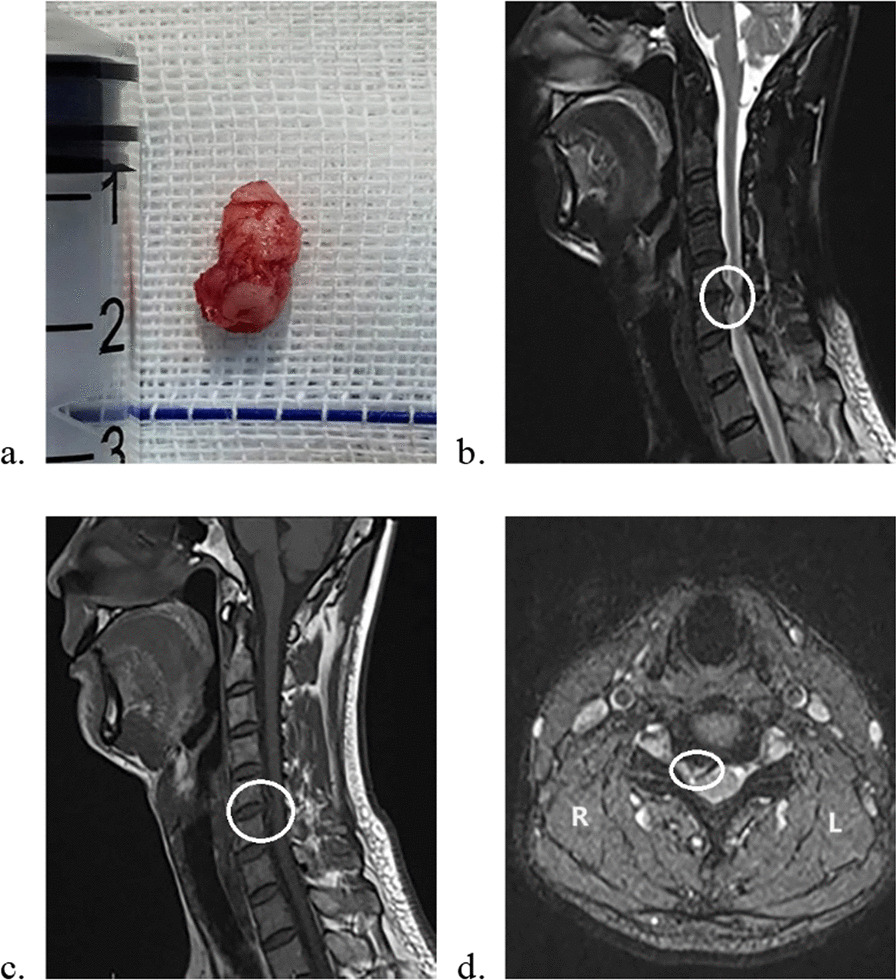
Sequestered disk in surgery. A 58-year-old woman complained of right-hand numbness adopted ACDF in our hospital. A sequestered disk was found in the anterior epidural space during the operation (**a**). The preoperative MRIs also showed an obvious cervical disk herniation at the C5-C6 level. *ACDF* anterior cervical decompression and fusion, *MRI* magnetic resonance imaging

This study was designed as a retrospective cohort study. A flow diagram of the study is shown in Fig. 1. The relevant data came from patients hospitalized in Shanghai Changzheng Hospital from May 2022 to December 2022. The patients were diagnosed with CDH and treated with ACDF, physical examinations and cervical MRI scans were normally used, and consistency between patients’ symptoms and medical examination was checked. Patients were at the age of 18 to 75 when they received surgery. Patients with the following coexistent diseases were excluded: cervical vertebral fracture, primary spinal stenosis, spinal tumor or infection, ossification of the posterior longitudinal ligament (OPLL) and acute spinal injury. ACDF was the only spinal operation they received; revision spinal surgery was excluded neither. Patients in the groups showed at least one symptom of cervical radiculopathy or myelopathy. Patients with myelopathy received surgery once diagnosed. For patients with mere cervical radiculopathy, a full evaluation was conducted in the outpatient department, patients with relatively mild symptoms or short symptom duration received conservative treatment, including nonsteroidal anti-inflammatory drugs, neurotrophic drugs and physical therapy. After at least 2 months of conservative treatment, surgery was considered in patients who were willing to accept operation because of unsatisfactory curative outcomes. Sequestered disks were exactly found during operation (Fig. 2). To decrease clinical heterogeneity, all operations were performed by the same spine surgeon (Dr. Tian). All the patients were ordered to wear semirigid cervical collars one month after the operation. Therefore, of the 130 patients we tracked, six patients were lost to follow-up, and 16 patients were excluded because of OPLL, posterior approach operation and revision surgery. Thus, 50 patients (46.3%) were included in the sequestered disk group, and 58 (54.7%) were included in the non-sequestered disk group. All patients were followed up for 6 months after the operation.

### Imaging studies

MRIs were completed at each patient according to a standardized protocol tailored to a 3Tesla scanner MRI. CTs and X-rays of the cervical spine were used to determine whether there were significant OPLL, giant osteophyte compression and primary spinal stenosis. The imaging results were independently interpreted by one radiologist and one spine surgeon, and a senior spine surgeon was consulted. However, intraoperative visual findings were definitively the most powerful evidence.

### Surgical technique and device description

**Fig. 3:**
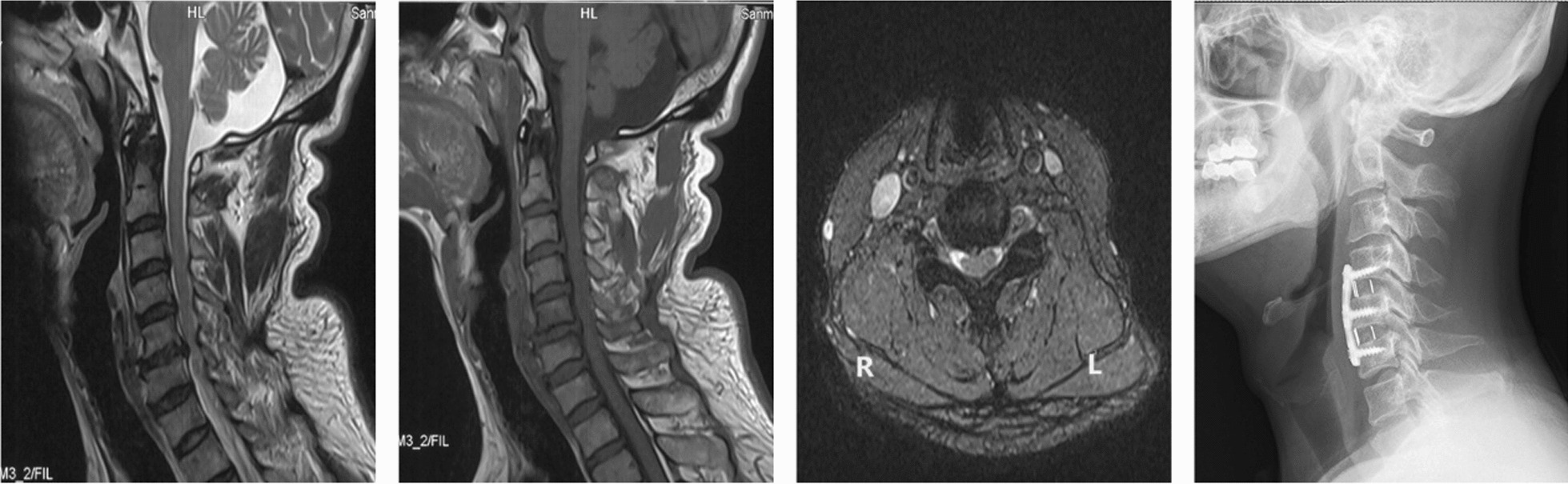
A classic case in the non-sequestered disk group. The preoperative MRIs showed cervical disk herniation at the C4-C6 level, the plain radiograph was taken two months after surgery. MRI: magnetic resonance imaging

**Fig. 4:**
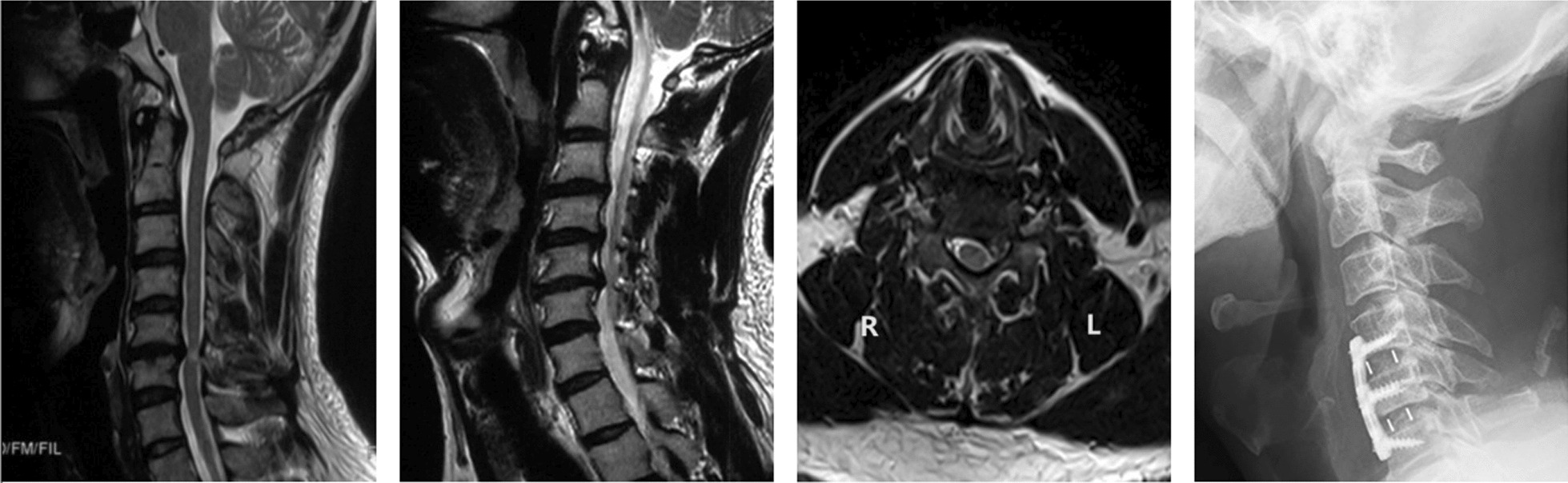
A classic case in the sequestered disk group. The preoperative MRIs showed a sequestered disk at the C5-C6 level, the plain radiograph was taken two months after surgery. MRI: magnetic resonance imaging

The patients received standard ACDF operation. The same type of implants was used in the surgery. During the surgery, patients were positioned supine on the operating table, received general anesthesia with endotracheal intubation. A right-sided horizontal surgical incision was located according to the anatomic landmarks. Platysma, superficial cervical fascia and deep cervical fascia were incised layer by layer through the Smith–Robinson approach. The trachea and esophagus were gently retracted to expose the prevertebral fascia. We used intraoperative fluoroscopy to confirm the correct level. Then, we carried out a complete discectomy and sufficient decompression of the nerve roots and spinal cord. The suitable poly ether ether ketone (PEEK) cages filled with autogenous bone were implanted in the disk space, titanium plate and screws (Venture, Medtronic Sofamor Danek USA, Memphis, TN) were fixed on the anterior surface of vertebral bodies(Figs.3 and 4). Finally, we generally placed a drain and closed incisions layer by layer. The drain was removed on postoperative day 1. After the operation, all the patients were ordered to wear semirigid cervical collars for one month.

### Evaluations

Prognosis assessments were conducted using the following scales: the Japanese Orthopedics Association (JOA) score, the Neck disability index (NDI) [9, 10] score and the Visual analog scale (VAS) [11] score. They are widely used in the study of cervical spondylosis. The subjects were interviewed face-to-face before the operation (defined as baseline; V1), and follow-up visits at the 2nd(V2), 4th(V3) and 6th(V4) months after the operation were undertaken. All evaluations were processed by the same doctor.

### JOA score (neck-version)

The JOA scale is widely used to evaluate the spinal cord function of patients with CDH. The total score is 17 points and consists of four aspects: upper limb function, lower limb function, sensory disorders and bladder function. The 1975 JOA score is the most appropriate version for assessing individuals from Asian populations (particularly those eating with chopsticks) [12]. The improvement in JOA scores was calculated as follows: (postoperative JOA score− preoperative JOA score)/(17 − preoperative JOA score) × 100% [13].

### NDI score

The NDI score was assessed on ten items: pain intensity, self-care, lifting, reading, headaches, concentration, work, driving, sleeping and recreation. Each item consists of six questions and is scored on a 0–5-point scale. The higher the score is, the greater the disability [14]. The improvement of NDI scores was calculated as follows: preoperative NDI score− postoperative NDI score.

### VAS score

The VAS score was announced by E.C Huskisson in 1974, which was widely used in pain assessment. The validity and reliability were tested by many scholars [15]. We marked figures from “0” to “10” equally in a 10-cm line, explained to the patients that “0” means no pain, “10” means unbearable pain, from “0” to “10,” the pain degrees gradually increased. The patients were asked to mark an appropriate score on the line that best represent their pain. The improvement in VAS scores was defined as follows: preoperative VAS score− postoperative VAS score.

### Statistical analyses

We used SPSS version 25 (IBM Corp, Armonk, NY, USA) to process all statistical analyses. All data were presented as mean and standard deviation (SD). We preferentially used parametric methods, as nonparametric methods tend to result in loss of information and decrease the validity of the tests. We used independent-samples t test to compare continuous data and Chi-squared test for categorical data between groups. Tests were two-tailed, and a *P*-value < 0.05 was considered statistically significant.

## Results

We analyzed the demographic data of the patients included in this study, which is shown in Table 1. There was no significant statistical difference in gender composition, average age, average course of disease or surgical segments between the two groups. However, preoperative cervical spine MRIs between the two groups were analyzed as follows: 32 patients in the sequestered disk group (64%) and 19 patients in the non-sequestered disk group (32.76%) presented with high signal intensity of the spinal cord on preoperative cervical T2-weighted MRI, with a significant difference (*P* < 0.001).

**Table 1:** Patient demographic details

Variable	Sequestered disk	Non-sequestered disk	*P*
Total, *n*	50	58	
Male, *n *(%)	29(58.00)	29(50.00)	0.406†
Mean age accepted ACDF, years(SD)	51.08 ± 10.58	51.96 ± 10.06	0.657*
Smoker, *n* (%)	15(30.00)	14(24.14)	0.493†
Diabetes, *n *(%)	5(10.00)	7(12.07)	0.733†
Mean duration of symptoms before ACDF, months (SD)	21.96 ± 42.26	33.20 ± 40.59	0.162*
Steps of ACDF, *n* (%)			0.947†
1	15(30.00)	16(27.59)	
2	24(48.00)	28(48.28)	
3	11(22.00)	14(24.14)	
High intensity onT2WI, *n *(%)	32(64.00)	19(32.76)	0.001†

*Independent-samples t test

^†^Chi-squared test

*SD* standard deviation, *ACDF* anterior cervical decompression and fusion

**Fig. 5:**
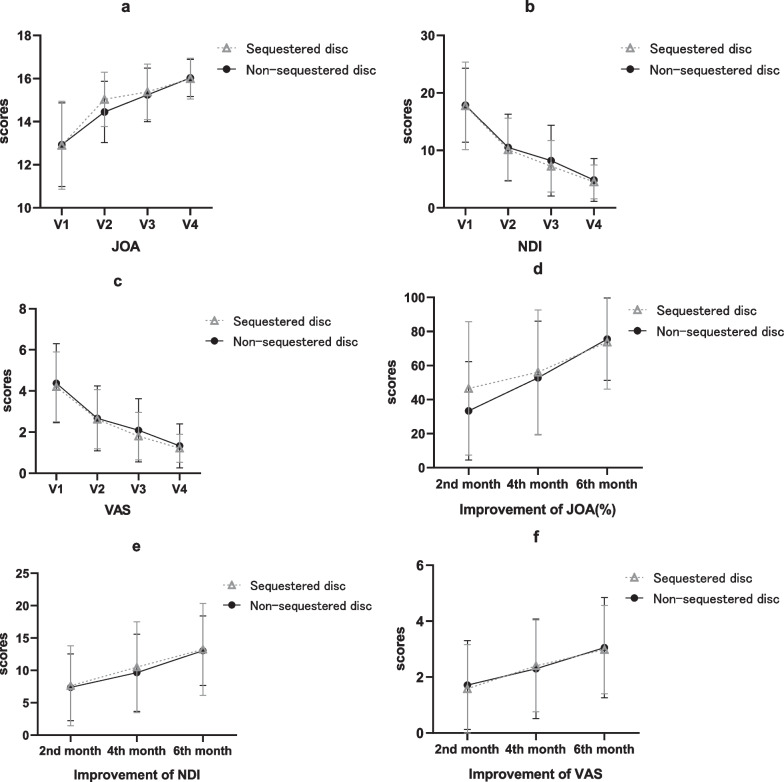
The changes in clinical outcomes. **a** JOA, **b** NDI, **c** VAS, **d** Improvement of JOA (%), **e** Improvement of NDI, **f** Improvement of VAS. *JOA* Japanese Orthopedics Association, *NDI* the Neck disability index, *VAS* the Visual analog scale. Improvement of JOA(%): (Vn − V1)/(17 − V1) × 100%; Improvement of NDI: V1 − Vn; Improvement of VAS: V1 − Vn

The clinical outcomes are presented in Tables 2,3,4 and Fig. 5. Compared with the preoperative values, the mean JOA, NDI and VAS scores in both groups were all improved after ACDF (*P* < 0.001). The mean JOA was 15.04 ± 1.26 in the sequestered disk group and was 14.45 ± 1.43 in the non-sequestered disk group two months after operation (*P* = 0.026 < 0.05). The improvement in JOA at two months after ACDF also showed a statistical difference: 46.58% ± 39.17% in the sequestered disk group and 33.39% ± 28.82% in the non-sequestered disk group (*P* = 0.047 < 0.05). There was no significant difference between the two groups in other parameters.

**Table 2:** Discrepancies of treatment effect between the two groups

	Sequestered disk	Non-sequestered disk	*P*
	*Baseline(V1)*		
Mean JOA(SD)	12.90 ± 2.04	12.93 ± 1.95	0.936
Mean NDI(SD)	17.76 ± 7.62	17.87 ± 6.44	0.930
Mean VAS(SD)	4.20 ± 1.70	4.38 ± 1.92	0.611
	*V2*		
Mean JOA(SD)	15.04 ± 1.26	14.45 ± 1.43	0.026
Mean NDI(SD)	10.12 ± 5.51	10.50 ± 5.79	0.729
Mean VAS(SD)	2.62 ± 1.44	2.67 ± 1.58	0.858
	*V3*		
Mean JOA(SD)	15.38 ± 1.29	15.24 ± 1.25	0.572
Mean NDI(SD)	7.24 ± 4.48	8.22 ± 6.17	0.352
Mean VAS(SD)	1.80 ± 1.16	2.09 ± 1.54	0.283
	*V4*		
Mean JOA(SD)	16.00 ± 0.95	16.03 ± 0.86	0.843
Mean NDI(SD)	4.50 ± 2.94	4.83 ± 3.74	0.618
Mean VAS(SD)	1.22 ± 0.68	1.33 ± 1.07	0.540

*JOA* Japanese Orthopedics Association, *NDI* the Neck disability index, *VAS* the Visual analog scale, *SD* standard deviation

Visits: baseline (V1); 2nd month after surgery (V2); 4th month after surgery (V3); 6th month after surgery (V4)

**Table 3:** Clinical outcomes in every group

	V1	V2	*P*(V2 − V1)	V3	*P* (V3 − V1)	V4	*P* (V4-V1)
*Sequestered disk*							
Mean JOA(SD)	12.90 ± 2.04	15.04 ± 1.26	< 0.001	15.38 ± 1.29	< 0.001	16.00 ± 0.95	< 0.001
Mean NDI(SD)	17.76 ± 7.62	10.12 ± 5.51	< 0.001	7.24 ± 4.48	< 0.001	4.50 ± 2.94	< 0.001
Mean VAS(SD)	4.20 ± 1.70	2.62 ± 1.44	< 0.001	1.80 ± 1.16	< 0.001	1.22 ± 0.68	< 0.001
*Non-sequestered disk*							
Mean JOA(SD)	12.93 ± 1.95	14.45 ± 1.43	< 0.001	15.24 ± 1.25	< 0.001	16.03 ± 0.86	< 0.001
Mean NDI(SD)	17.87 ± 6.44	10.50 ± 5.79	< 0.001	8.22 ± 6.17	< 0.001	4.83 ± 3.74	< 0.001
Mean VAS(SD)	4.38 ± 1.92	2.67 ± 1.58	< 0.001	2.09 ± 1.54	< 0.001	1.33 ± 1.07	< 0.001

*JOA* Japanese Orthopedics Association, *NDI* the Neck disability index, *VAS* the Visual analog scale, SD: standard deviation. Visits: baseline (V1); 2nd month after surgery (V2); 4th month after surgery (V3); 6th month after surgery (V4)

**Table 4:** The improvement of clinical outcomes at six-month follow-up

	Sequestered disk	Non-sequestered disk	*P*
*2nd month PO*			
Improvement of JOA(%)	46.58 ± 39.17	33.39 ± 28.82	0.047
Improvement of NDI	7.64 ± 6.20	7.38 ± 5.18	0.812
Improvement of VAS	1.58 ± 1.58	1.71 ± 1.59	0.679
*4th month PO*			
Improvement of JOA(%)	56.13 ± 36.59	52.80 ± 33.36	0.626
Improvement of NDI	10.52 ± 7.03	9.66 ± 5.96	0.490
Improvement of VAS	2.40 ± 1.64	2.30 ± 1.78	0.747
*6th month PO*			
Improvement of JOA(%)	73.80 ± 27.59	75.52 ± 24.15	0.728
Improvement of NDI	13.26 ± 7.11	13.05 ± 5.37	0.863
Improvement of VAS	2.98 ± 1.58	3.05 ± 1.79	0.827

Improvement of JOA (%): (Vn − V1)/(17 − V1) × 100%; Improvement of NDI: V1-Vn; Improvement of VAS: V1-Vn. *JOA* Japanese Orthopedics Association, *NDI* the Neck disability index, *VAS* the Visual analog scale. *PO* post-operation

## Discussion

Based on a comprehensive literature search, we found that studies on CDH mainly focused on the surgical necessity and efficacy of conservative treatment, the choice of surgical approach and method, the reports of rare diseases such as intradural cervical disk herniation and the clinical significance of high signal intensity of spinal cord changes on T2-weighted MRI caused by CDH. Yang et al. [16] studied the different classifications and clinical results of CDH shown in MRI examination and found that in patients with cervical radiculopathy, the type of intervertebral disk herniation before surgery was not related to the severity of symptoms, and the surgical improvement was also not related to the pathological type. However, Yang's research on CDH focused on bulge and herniation and did not fully reflect the sequestered type. Articles about the postoperative efficacy of each pathological classification of CDH have not been well published. Therefore, we may study this aspect for the first time.

In this study, the two groups of patients generally achieved obvious symptom improvement and functional recovery within six months after surgery, which was consistent with the mainstream in current research [17]. Preoperative symptom assessment was consistent between the two groups, while the JOA scores and improvement rates both showed significant difference two months after surgery. We also observed whether patients with sequestered cervical disks tended to have a high signal intensity in the spinal cord on the preoperative T2-weighted MRI, which seemed to be a significant difference in the cases we collected.

As the largest avascular tissue in human body, pathological and histological studies of the intervertebral disks have been hot topics for quit a long time. Sally et al. [18] found that the morphology of disk herniations can be very heterogeneous and may include tissue of nucleus pulposus, annulus fibrosus or cartilage end plate. Therefore, the sequestered cervical disk is not just the nucleus pulposus but may contain other tissue [19]. Sequestered cervical disk re-establishes contact with the internal environment, which may lead to a series of inflammatory immune responses, neovascularization and free nerve fibers development [20], which seems more likely to lead to more severe symptoms than mere mechanical compression. From the perspective of the progression of disk herniation, whether the sequestered disk is the end in the process of chronic intervertebral disk herniation or just a subcategory caused by external stimulation or other factors in the process of herniation, has attracted increasing in-depth research.

We also compared preoperative MRI with the surgical findings (Fig. 2). The sequestered fragments usually exhibit high signal intensity on T2WI due to the relatively high-water content of the free nucleus pulposus, and T1WI is also crucial in detecting sequestered fragments that sequestered disks maintain moderate to low intensity. Some typical sequestered disks present as posterior migrating fragments cling to the adjacent vertebrae. In the setting of CDH, intramedullary signal changes are generally observed in MRI, indicating pathophysiological processes such as edema, demyelination and structural changes [21]. All the signal changes in the sequestered disk group are at the spinal cord segment compressed by the sequestered fragments. The sequestered disk often leads to a higher canal-occupying ratio, which means more physical compression of the spinal canal contents, immune reactions or chemical stimulation may further affect the spinal cord of the corresponding segments. Thus, degeneration, edema and necrosis in spinal cord cells may be more serious and more common in patients with sequestered disks, leading to intramedullary signal changes in MRI. However, in-depth study on the relationship between sequestered cervical disks and high signal intensity on spinal MRI remain lacking. The relevancy of cervical intramedullary signal changes in MRI and prognosis has been extensively researched. Yagi et al. [22] reported that long-term clinical outcome was significantly worse in patients with intramedullary signal intensity changes on MRI. A global cohort study conducted by Nouri et al. [23] pointed out that the presence of T2WI-hyperintensity in isolation adds little to the clinical picture. The mainstream theory indicates that intramedullary signal changes suggest cavitation or necrosis in the spinal cord, which is associated with poorer prognosis [24]. However, according to our follow-up results, patients with sequestered disks also achieved good results within 6 months after ACDF, even though the signal changes were more common in the sequestered disk group.

In the patients with sequestered disks we tracked, the sequestered disk was basically located in the anterior epidural space, and no case broke through the dural sac. Cases with intradural cervical herniation have been reported by some scholars [25]. Meanwhile, cases of spontaneous regression of herniated disks have been widely reported with considerable efficacy of conservative treatment, but the process may be quite long [26]. However, related reports and researches have mainly focused on the lumbar disk [27, 28]. Some scholars have carried out mechanism research on this problem. In a recent review, Yu et al. [29] summarized the biological mechanisms involved in the phenomenon of LDH resorption and highlighted the critical role of autoimmune responses in spontaneous disk resorption, including inflammatory responses mediated by macrophage infiltration interacting with the disk, enzymatic degradation responses and angiogenesis, specifically mentioning that sequestration of LDH could be a favorable factor in LDH resorption. However, the analysis of relevant mechanisms is still in the exploratory stage. At the same time, compared with the lumbar spine, spontaneous resorption in the cervical disk has seldomly been reported [30]. Related mechanism studies reported in CDH resorption were more of the same [31]: release of basic fibroblast grow factors, endothelial cell proliferation, chemotaxis of inflammatory cells into the disk fragment and foreign body inflammatory reaction. In contrast, functional impairments of the cervical spinal cord and nerve roots are more unacceptable to patients than LDH. Therefore, early surgical intervention is still reasonable and necessary rather than relying on the small probability of CDH regression. Studies on the prognosis of LDH have also mentioned that patients with sequestered LDH often benefit greatly from surgery [32]. Therefore, we prefer early surgical treatment in people with sequestered cervical disk. Such patients in our hospital usually adopt ACDF, and there were clear conclusions about the efficacy of ACDF [33, 34], which were also confirmed by our results.

The study also has limitations, one being the sample source, single-center collection may result in unconvincing outcomes, while small sample size leads to an increased risk of a type II error. Another limitation is the short follow-up time. From the perspective of clinical practice, a longer follow-up time can often obtain more sufficient data for research and judgment. Finally, the symptoms of some patients are often atypical, and some information may be lost after quantitative rating, which results in inaccurate assessment.

## Conclusion

Patients with sequestered cervical disks who received ACDF seemed to have a higher degree of symptom improvement two months after surgery than those without sequestered cervical disks. However, the preoperative symptoms and long-term postoperative effect of both groups were basically consistent, and both achieved good postoperative benefits. In addition, CDH with sequestered disks appears to be more likely to cause high signal intensity changes in the compressed cervical spine on T2-weighted MRI. Therefore, we prefer early positive surgery in patients with sequestered cervical disks from the clinical point of view.

## Data Availability

The datasets generated and analyzed during the current study are available from the corresponding author on reasonable request.

## Abbreviations

CDH: Cervical disk herniation
LDH: Lumbar disk herniation
MRI: Magnetic resonance imaging
ACDF: Anterior cervical decompression and fusion
OPLL: Ossification of the posterior longitudinal ligament
CT: Computed tomography
VAS: Visual analog scale
JOA: Japanese Orthopedics Association
NDI: Neck disability index
SD: Standard deviation

## References

[1] Roh JS, Teng AL, Yoo JU, Davis J, Furey C, Bohlman HH (2005). Degenerative disorders of the lumbar and cervical spine. Orthop Clin North Am.

[2] Sucuoglu H, Barut AY (2021). Clinical and radiological follow-up results of patients with sequestered lumbar disc herniation: a prospective cohort study. Med Princ Pract.

[3] Fardon DF, Williams AL, Dohring EJ, Murtagh FR, Gabriel Rothman SL, Sze GK (2014). Lumbar disc nomenclature: version 2.0 recommendations of the combined task forces of the North American Spine Society, the American Society of Spine Radiology, and the American Society of Neuroradiology. Spine (Phila Pa 1976).

[4] Khil EK, Choi I, Lee SA, Seo W, Choi JA (2023). Novel MRI signs of ruptured disc in the cervical spine with intraoperative comparisons. Eur Radiol.

[5] Saal JS, Saal JA, Yurth EF (1996). Nonoperative management of herniated cervical intervertebral disc with radiculopathy. Spine (Phila Pa 1976).

[6] Chin-See-Chong TC, Gadjradj PS, Boelen RJ, Harhangi BS (2017). Current practice of cervical disc arthroplasty: a survey among 383 AOSpine International members. Neurosurg Focus.

[7] Mazas S, Benzakour A, Castelain JE, Damade C, Ghailane S, Gille O (2019). Cervical disc herniation: Which surgery?. Int Orthop.

[8] Sahai N, Changoor S, Dunn CJ, Sinha K, Hwang KS, Faloon M, Emami A (2019). Minimally invasive posterior cervical foraminotomy as an alternative to anterior cervical discectomy and fusion for unilateral cervical radiculopathy: a systematic review and meta-analysis. Spine (Phila Pa 1976).

[9] Vernon H (2008). The neck disability index: state-of-the-art, 1991–2008. J Manipul Physiol Ther.

[10] Vernon H, Mior S (1991). The neck disability index: a study of reliability and validity. J Manip Physiol Ther.

[11] Dixon JS, Bird HA (1981). Reproducibility along a 10 cm vertical visual analogue scale. Ann Rheum Dis.

[12] Furlan JC, Catharine Craven B (2016). Psychometric analysis and critical appraisal of the original, revised, and modified versions of the Japanese Orthopaedic Association score in the assessment of patients with cervical spondylotic myelopathy. Neurosurg Focus.

[13] Hirabayashi K, Miyakawa J, Satomi K, Maruyama T, Wakano K (1981). Operative results and postoperative progression of ossification among patients with ossification of cervical posterior longitudinal ligament. Spine (Phila Pa 1976).

[14] Kalsi-Ryan S, Singh A, Massicotte EM, Arnold PM, Brodke DS, Norvell DC, Hermsmeyer JT, Fehlings MG (2013). Ancillary outcome measures for assessment of individuals with cervical spondylotic myelopathy. Spine (Phila Pa 1976).

[15] Maxwell C (1978). Sensitivity and accuracy of the visual analogue scale: a psycho-physical classroom experiment. Br J Clin Pharmacol.

[16] Yang X, Arts MP, Bartels R, Vleggeert-Lankamp CLA (2022). The type of cervical disc herniation on MRI does not correlate to clinical outcomes. Bone Joint J.

[17] Zhang AS, Myers C, McDonald CL, Alsoof D, Anderson G, Daniels AH (2022). Cervical myelopathy: diagnosis, contemporary treatment, and outcomes. Am J Med.

[18] Roberts S, Evans H, Trivedi J, Menage J (2006). Histology and pathology of the human intervertebral disc. J Bone Joint Surg Am.

[19] Brock M, Patt S, Mayer HM (1992). The form and structure of the extruded disc. Spine (Phila Pa 1992).

[20] Kokubo Y, Uchida K, Kobayashi S, Yayama T, Sato R, Nakajima H, Takamura T, Mwaka E, Orwotho N, Bangirana A (2008). Herniated and spondylotic intervertebral discs of the human cervical spine: histological and immunohistological findings in 500 en bloc surgical samples. Laboratory investigation. J Neurosurg Spine.

[21] Nouri A, Martin AR, Mikulis D, Fehlings MG (2016). Magnetic resonance imaging assessment of degenerative cervical myelopathy: a review of structural changes and measurement techniques. Neurosurg Focus.

[22] Yagi M, Ninomiya K, Kihara M, Horiuchi Y (2010). Long-term surgical outcome and risk factors in patients with cervical myelopathy and a change in signal intensity of intramedullary spinal cord on magnetic resonance imaging. J Neurosurg Spine.

[23] Nouri A, Martin AR, Kato S, Reihani-Kermani H, Riehm LE, Fehlings MG (2017). The relationship between MRI signal intensity changes, clinical presentation, and surgical outcome in degenerative cervical myelopathy: analysis of a global cohort. Spine (Phila Pa 1976).

[24] Badhiwala JH, Ahuja CS, Akbar MA, Witiw CD, Nassiri F, Furlan JC, Curt A, Wilson JR, Fehlings MG (2020). Degenerative cervical myelopathy: update and future directions. Nat Rev Neurol.

[25] Pan J, Li L, Qian L, Teng H, Shen B, Tan J, Zhou W, Yang M (2011). Intradural cervical disc herniation: report of two cases and review of the literature. Spine (Phila Pa 1976).

[26] Kobayashi N, Asamoto S, Doi H, Ikeda Y, Matusmoto K (2003). Spontaneous regression of herniated cervical disc. Spine J.

[27] Karavelioglu E, Eser O, Sonmez MA (2013). Spontaneous resorption of sequestrated lumbar disc fragment. Spine J.

[28] Ushewokunze S, Abbas N, Dardis R, Killeen I (2008). Spontaneously disappearing lumbar disc protrusion. Br J Gen Pract.

[29] Yu P, Mao F, Chen J, Ma X, Dai Y, Liu G, Dai F, Liu J (2022). Characteristics and mechanisms of resorption in lumbar disc herniation. Arthritis Res Ther.

[30] Kocyigit F, Kocyigit A, Manisali M, Akalin E (2011). Resorption of a sequestered cervical disc confirmed by magnetic resonance imaging: long term follow-up. Case report. Eur J Phys Rehabil Med.

[31] Vinas FC, Wilner H, Rengachary S (2001). The spontaneous resorption of herniated cervical discs. J Clin Neurosci.

[32] Kerr D, Zhao W, Lurie JD (2015). What are long-term predictors of outcomes for lumbar disc herniation? A randomized and observational Study. Clin Orthop Relat Res.

[33] Landers MR, Addis KA, Longhurst JK, Vom Steeg BL, Puentedura EJ, Daubs MD (2013). Anterior cervical decompression and fusion on neck range of motion, pain, and function: a prospective analysis. Spine J.

[34] Kim HJ, Choi BW, Park J, Pesenti S, Lafage V (2019). Anterior cervical discectomy and fusion can restore cervical sagittal alignment in degenerative cervical disease. Eur J Orthop Surg Traumatol.

